# Oral maxillofacial neoplasms in an East African population a 10 year retrospective study of 1863 cases using histopathological reports

**DOI:** 10.1186/1472-6831-8-19

**Published:** 2008-07-23

**Authors:** Adriane Kamulegeya, Boniphace M Kalyanyama

**Affiliations:** 1 Dept of Dentistry, Faculty of Medicine, Makerere University, box 7072, Kampala, Uganda; 2 Department of Oral Surgery and Oral Pathology, School of Dentistry, Muhimbili University of Health and Allied Sciences (MUHAS) Dar es Salaam, Tanzania

## Abstract

**Background:**

Neoplasms of the oral maxillofacial area are an interesting entity characterized by differences in nomenclature and classification at different centers.

We report neoplastic histopathological diagnoses seen at the departments of oral maxillofacial surgery of Muhimbili and Mulago referral hospitals in Tanzania and Uganda respectively over a 10-year period.

**Methods:**

We retrieved histopathological reports archived at the departments of oral maxillofacial surgery of Muhimbili and Mulago referral hospitals in Tanzania and Uganda respectively over a 10-year period from June 1989–July 1999.

**Results:**

In the period between June 1989 and July 1999, 565 and 1298 neoplastic oro-facial cases were retrieved of which 284 (50.53%) and 967 (74.54%) were malignant neoplasms at Muhimbili and Mulago hospitals respectively. Overall 67.28% of the diagnoses recorded were malignant with Kaposi's sarcoma (21.98%), Burkiits lymphoma (20.45%), and squamous cell carcinoma (15.22%) dominating that group while ameloblastoma (9.23%), fibromas (7.3%) and pleomorphic adenoma (4.95%) dominated the benign group.

The high frequency of malignancies could be due to inclusion criteria and the clinical practice of selective histopathology investigation. However, it may also be due to higher chances of referrals in case of malignancies.

**Conclusion:**

There is need to reexamine the slides in these two centers in order to bring them in line with the most recent WHO classification so as to allow for comparison with reports from else where.

## Background

Neoplasms of the oral maxillofacial area are a source of great differences in nomenclature and classification making comparison from different countries difficult. Reports from different parts of the world show differences in the pattern of maxillofacial tumors seen. [1-5] A couple of authors have revisited the histopathological slides of certain groups of maxillofacial tumors in their centers with the aim of reclassifying the diagnoses made at the time to bring them in line with the current WHO classification. [6,7] This is meant to bring sanity in the body of research and clinical records. Uganda at the moment has only one serving oral pathologist and Tanzania is no better, hence reliance on already over-stretched general pathologists to do the oral histopathological diagnosis. The burden of tropical infectious diseases such as malaria, tuberculosis and HIV/AIDS relegates oro-facial diseases to the bottom of the priority list by both government and donor agencies.

Although some work has been reported on these neoplasms from East Africa, most was done years back and the numbers reported were few.

In 1956, Pritam and Cook reported sarcomas, adamantinoma and carcinomas as the commonest oral maxillofacial neoplasms in Uganda. [8] Four years later Dodge reported an unusually high incidence of odontogenic tumors in Ugandan Africans. [9] However, when Slavin and Cameron reported on ameloblastoma from both Uganda and Tanzania they attributed the high numbers to a harvesting period. [10] Recently Simon et al reported that some of the tumors that were initially thought to have a racial preponderance for African blacks are in fact equal in incidence with other parts of the world. [11] Although Wakiaga J.M et al in a report from Kenya had high number of cases, they restricted inclusion to cases of jaw tumors only. Ameloblastoma, Burkitts lymphoma, ossifying fibroma and osteogenic sarcoma were reported as being the commonest in that order. [12]

We felt that an update on the different histopathological entities reported and recorded at the oral maxillofacial surgery departments in East Africa was long overdue. We hope it will show the huge discrepancies in classification with the standing WHO classification and as a result generate interest and funding so as to streamline our records.

## Methods

Mulago Hospital and Muhimbili National Referral Hospital are the two biggest referral hospitals for Uganda and Tanzania respectively. They were the main treatment centers for oral maxillofacial neoplasms until recently when some other regional referral centers in Tanzania got the personnel to man the departments. They both house the national cancer registry at the departments of human pathology and morbid anatomy. The normal procedure is that the histopathological results are filed at both the relevant departments as well as the department of human pathology and morbid anatomy.

The data presented here was obtained from the biopsy results files in the departments of oral maxillofacial surgery and oral pathology at both hospitals stretching from June 1989 to July 1999. The choice of the time interval was due to the fact that from the year 2000 some regional referral hospitals in Tanzania had set up oral and maxillofacial surgery departments hence reducing the catchment area for Muhimbili hospital. The tumor type, age, sex and site of lesions were recorded in a pre-designed data collection form. During data collection, the diagnoses were recorded as reported on the biopsy result slips. However, "unspecified" such as undetermined malignancies and those that gave histological characteristics but no final diagnoses were excluded from the study during analysis. Reports of fine needle aspiration cytology and microscopy of blood or fluid smears were excluded. Repeat biopsies were counted once with the later diagnosis taken. This was in light of the fact that at both centers the previous biopsy number is recorded when a repeat is done allowing the pathologists to review the old slide alongside the repeat slide enabling a more informed decision. Those repeat biopsies that were done due to recurrence after treatment were recorded once except if the second time around a different diagnosis was given. Non neoplastic cysts, dysplastic and inflammatory lesions were excluded from the study.

An interval of 10 years was used to classify the data into age groups. As for those that were not specified or recorded as adult, they were categorized as unknown while the ones recorded as child were placed under the 10 years and below.

The site of the neoplasm was taken as recorded on the slips. However, those cases appearing adjacent to major salivary glands should be taken with caution. The lip included both lower and upper while the palate represented hard and soft palate. Site recorded as oral cavity the neoplasm involved the tongue (oral and oropharygeal parts), floor of the mouth, gingival and buccal mucosa.

Statistical analysis including student's t tests and simple proportion tests done using excel Microsoft office 2003.

## Results

In the period between June 1989 and July 1999, 565 and 1298 neoplastic oro-facial cases were retrieved of which 284 (50.53%) and 967 (74.54%) were malignant neoplasms at Muhimbili and Mulago hospitals respectively. In Tanzania (Muhimbili hospital), squamous cell carcinoma (32.75%) was the commonest in the malignant category followed by Burkitts lymphoma (32.40%) and Kaposis sarcoma (15.14%). Only one case of malignant odontogenic neoplasm (malignant ameloblastoma) was reported.

On the other hand, in Uganda (Mulago hospital), Kaposis sarcoma(37.78%) was the commonest malignant neoplasm followed by Burkitts lymphoma (30,12%)then squamous cell carcinoma (19.67%). Combined data (i.e Muhimbili plus Mulago), malignant neoplasms were 67.28% of all retrieved results with Kaposis sarcoma (32.64%), Burkitts lymphoma (30.56%) and squamous cell carcinoma (22.64%) dominating.

As for the benign neoplasms, ameloblastoma (35.61%), pleomorphic adenoma (17.63%) and fibromas (17.57%) were the most frequent in Tanzania while in Uganda it was fibromas (26.67%), ameloblastoma (22.12%), pleomorphic adenoma (13.03%) and papilloma (10.6%). The different diagnoses seen at the two hospitals are shown in Table 1. Overall, odontogenic neoplasms were 11.73%, 9.8% (Uganda) 15.30% (Tanzania).

**Table 1:** Distribution of oralfacial neoplasms in Mulago Uganda (UG) and Muhimbili Tanzania (TZ)

Diagnosis	Abbreviation	UG	TZ	% of overall
Ameloblastoma	AME	73	99	9.23
Odontogenic myxoma	MYX	6	15	1.13
Ameloblastic fibroma	AMEF	1	3	0.21
Adenomatoid odontogenic tumour	AOT	1	1	0.11
Odontoma	ODT	1	0	0.05
Odontogenic fibroma	ODF	0	5	0.27
Malignant ameloblastoma	MAME	0	1	0.05
				
Fibroma	FIB	88	48	7.30
Ossifying fibroma	OF	0	2	0.11
Cementifying fibroma	CF	4	5	0.48
Chondroma	CHD	1	2	0.16
Osteoma	OST	5	4	0.48
				
Osteo sarcoma	OSS	2	5	0.38
Fibro sarcoma	FIS	4	1	0.27
Chondrosarcoma/Chondro myxoid fibro sarcoma	CHS	7	1	0.43
Myxoid sarcoma	MYXS	1	0	0.05
Round cell carcinoma	RCS	0	1	0.05
				
Burkitts lymphoma	BUR	291	91	20.54
Non Hodgkin's lymphoma	NHL	11	6	0.91
Histiocytic lymphoma	HLH	1	0	0.05
Centroblastic polymorphic lymphoma	CPL	0	1	0.05
				
Squamous cell carcinoma	SCCa	190	93	15.22
Anaplastic carcinoma	ACa	9	8	0.86
Un/Poorly differentiated carcinoma	UCa	19	0	1.02
Nasopharyngeal carcinoma	NPCa	1	0	0.05
Non keratinizing carcinoma of tonsil	NKTca	0	1	0.05
Verrucous carcinoma	Vca	3	3	0.32
Bassal cell carcinoma	BCCa	0	1	0.05
				
Adenoma	ADN	16	3	1.02
Pleomorphic adenoma	PLA	43	49	4.95
Monomorphic adenoma	MOA	0	1	0.05
Oxyphill cell adenoma	OCA	3	0	0.16
Adeno carcinoma	ADCa	22	10	1.72
Adenocystic carcinoma (Cylindroma)	ACCa	14	5	1.02
Salivary gland carcinoma	SGCa	2	1	0.16
Mucous secreting carcinoma	MSCa	4	0	0.22
Muco epidermoid carcinoma	MECa	5	4	0.48
Carcinoma ex pleomorphic adenoma	CaPLA	1	0	0.05
				
Haemagio pericytoma	HAP	2	2	0.22
Kaposis sarcoma	KS	365	44	21.98
Haemagio sarcoma	HAS	0	1	0.05
				
Granular cell myoblastoma	GCM	6	0	0.32
Leiomyoma	LEI	0	1	0.05
Rhabdomyosarcoma	RHS	8	1	0.48
				
Lymphagioma	LPH	11	1	0.65
Lymphagiosarcoma	LPHS	0	1	0.05
				
Lipoma	LIP	19	10	1.56
Liposarcoma	LIS	1	1	0.11
				
Melanotic prognoma	MEP	1	0	0.05
Melanoma	MEL	5	2	0.38
				
Hamartoma	HAM	4	2	0.32
Papilloma	PAP	35	22	3.06
Neurofibroma	NEF	10	2	0.65
Schwarnoma	SCH	0	2	0.11
Mesenchynoma	MSA	0	1	0.05
Calcifying Epithelioma of Malherbe	CEM	1	1	0.11
Chodro Syrigoma	CHSY	1	0	0.05
Retinoblastoma	REBA	0	1	0.05

**TOTAL**		**1298**	**565**	

The gender distribution of the different neoplasms is as shown in Table 2. Of the retrieved cases 53.71% were males compared to 45.32% females and 0.97% were not specified. The over all the male female ratio was 1.2:1, 1.3:1 in Tanzania compared to 1.14:1 in Uganda. Of the male cases 71.74% were malignant compared to 61.88% among females.11.12% of the male neoplasms were odontogenic compared to 21.65% of the female cases.

**Table 2:** Gender distribution of the oral facial neoplasms

Abbreviation	Females	Males	Unspecified	Total	**M:F Ratio**	
					
					UG	TZ
AME	73	97	2	172	1.28:1	1.36:1
MYX	15	4	2	21	*	1:2.25
AMEF	4	-	-	4	*	*
AOT	1	1	-	2	**	*
ODT	1	-	-	1	-	*
ODF	3	2	-	5	-	1:1.5
MAME	1	-	-	1	-	*
						
FIB	79	57	-	136	1:1.32	1:1.53
OF	2	-	-	2	-	*
CF	6	3	-	9	*	1:2
CHD	1	2	-	3	**	1:1
OST	1	8	-	9	**	3:1
						
OSS	2	5	-	7	1:1	4:1
FIS	4	1	-	5	*	**
CHS	2	6	-	8	1:6	*
MYXS	-	1	-	1	**	-
RCS	-	1	-	1	-	**
						
BUR	252	128	2	382	1.89:1	2.25:1
NHL	5	12	-	17	1.75:1	1:1
HLH	1	-	-	1	*	-
CPL	-	1	-	1	-	**
						
SCCa	126	153	4	283	1:1.01	1.9:1
ACa	9	8	-	17	1:1.25	1:1
UCa	6	13	-	19	2.2:1	-
NPCa	1	-	-	1	*	-
NKTca	-	1	-	1	-	**
Vca	1	5	-	6	**	2:1
BCCa	1	-	-	1	-	*
						
ADN	10	8	1	19	1:1	*
PLA	51	41	-	92	1:1.3	1:1.2
MOA	1	-	-	1	-	*
OCA	3	-	-	3	*	-
ADCa	15	17	-	32	1:1.2	1:1
ACCa	10	7	2	19	1:1.6	1:1
						
SGCa	1	2	-	3	1:1	**
MSCa	1	3	-	4	3:1	-
MECa	2	7	-	9	1.5:1	**
CaPLA	-	-	1	1	-	-
						
HAP	1	3	-	4	**	1:1
KS	199	208	2	409	1.1:1	1:1
HAS	1	-	-	1	-	*
						
GCM	5	1	-	6	1:2.5	-
LEI	1	-	-	1	-	*
RHS	2	7	-	9	3:1	**
						
LPH	7	5	-	12	1:1.75	**
LPHS	-	1	-	1	-	**
						
LIP	13	15	1	29	1:1.57	4:1
LIS	1	1	-	2	*	**
						
MEP	-	1	-	1	**	-
MEL	4	3	-	7	1:4	**
						
HAM	2	4	-	6	3:1	1:1
PAP	36	20	1	57	1:1.27	1:1.4
NEF	6	6	-	12	1:1.5	**
SCH	2	-	-	2	-	*
MSA	-	1	-	1	-	**
CEM	2	-	-	2	*	*
CHSY	1	-	-	1	*	-
REBA	-	1	-	1	-	**

**TOTAL**	974	871	18	1863		

**Figure 1:**
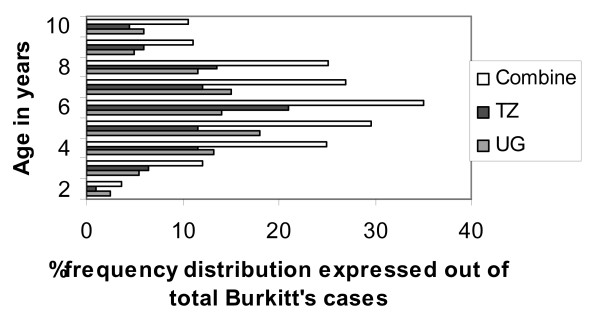
% distribution of Burkkit's lyphoma for those 10 years and below.

Age distribution of the different diagnosis is shown in table 3. The overall average age was 29.29 ± 19.72 with a range of 0.06–97 years. The neoplasms showed a wide range of age distribution with most neoplasms peaking in the second and third decade except Burkitts lymphoma that peaked below 10 years (Figure 1). Squamous cell carcinoma peaked in the sixth decade and dominated malignancies in the sixth decade upwards.

**Table 3:** Combined age distribution of oral facial neoplasms biopsy results l

***Dx***	***0–10***	***11–20***	***21–30***	***31–40***	***41–50***	***51–60***	***61–70***	***71–80***	***81–90***	***91***^+^	***US***	***mean***	***Range***
AME	2	36	54	33	14	11	3	1	-	-	18	30.52	5–75
MYX	5	4	5	1	1	1	1	-	-	-	3	22.42	6–64
AMEF	1	1	1	1	-	-	-	-	-	-	-	27.67	15–38
AOT	-	1	-	-	-	-	-	-	-	-	1	*	*
ODT	-	-	1	-	-	-	-	-	-	-	-	*	*
ODF	-	3	2	-	-	-	-	-	-	-	-	19.80	14–25
MAME	-	-	-	1	-	-	-	-	-	-	-	*	*
													
FIB	18	30	22	22	21	11	11	-	-	-	1	31.00	0.5–70
OF											2		
CF	-	3	4	-	-	1	-	-	-	-	1	27.00	12–52
CHD	-	-	1	1	-	1	-	-	-	-	-	40.50	30–58
OST	2	2	4	-	-	-	-	-	-	-	1	21.60	8–41
													
OSS	-	1	1	1	3	-	-	-	-	-	1	35.20	20–45
FIS	1	1	-	2	-	-	-	-	-	-	1	26.00	6–40
CHS	-	1	2	3	1	-	-	-	-	-	1	30.71	14–49
MYXS	-	-	-	-	1	-	-	-	-	-	-	*	*
RCS	-	1	-	-	-	-	-	-	-	-	-	*	*
													
BUR	341	35	2	-	1	-	-	-	-	-	3	7.21	2–45
NHL	2	5	1	1	2	2	4	-	-	-	-	35.33	1.5–63
HLH	-	-	-	1	-	-	-	-	-	-	-	*	*
CPL	-	-	-	1	-	-	-	-	-	-	-	*	*
													
SCCa	1	2	18	28	56	78	51	20	4	2	23	*	*
ACa	2	1	3	1	2	3	-	2	-	-	3	54.50	18–80
UCa	-	-	2	4	5	3	2	2	-	-	1	49.22	23–72
NPCa	-	-	1	-	-	-	-	-	-	-	-	*	*
NKTca	-	-	-	-	1	-	-	-	-	-	-	*	*
Vca	-	-	1	1	-	3	-	-	1	-	-	53.84	22–80
BCCa	-	-	-	-	-	1	-	-	-	-	-	*	*
													
ADN	2	3	7	2	2	1	1	-	-	-	1	29.16	3.1–61
PLA	3	14	30	19	9	6	6	1	-	-	4	32.58	5–71
MOA	-	-	-	-	1	-	-	-	-	-	-	*	*
OCA	-	3	-	-	-	-	-	-	-	-	-	19.00	17–20
ADCa	-	2	6	6	4	9	2	3	-	-	-	46.78	18–80
ACCa	-	3	2	1	6	4	1	1	-	-	1	46.86	20–65
													
SGCa	-	-	-	-	1	2	-	-	-	-	-	52.50	47–60
MSCa	-	1	-	2	1	-	-	-	-	-	-	33.00	14–48
MECa	-	2	1	1	2	2	-	-	-	-	1	37.22	13–60
CaPLA	-	-	-	-	-	-	-	-	-	-	1	*	*
													
HAP	1	2	-	-	-	-	-	1	-	-	-	28.50	7–80
KS	30	32	176	109	34	11	4	-	-	-	13	31.78	1.6–68
HAS	-	-	-	-	-	-	-	-	-	-	1	*	*
													
GCM	4	-	1	-	1	-	-	-	-	-	-	12.93	.06–49
LEI	-	1	-	-	-	-	-	-	-	-	-	*	*
RHS	3	4	1	-	1	-	-	-	-	-	-	*	*
													
LPH	6	2	1	-	1	-	2	-	-	-	-	17.61	0.7–70
LPHS	-	-	1	-	-	-	-	-	-	-	-	*	*
													
LIP	3	7	6	2	2	3	-	-	2	-	4	33.30	1–88
LIS	-	-	-	1	-	1	-	-	-	-	-	51.50	36–67
													
MEP	1	-	-	-	-	-	-	-	-	-	-	*	*
MEL	-	-	-	1	-	1	2	1	-	-	2	60.60	40–72
													
HAM	2	3	-	-	-	-	1	-	-	-	-	18.00	5–70
PAP	16	18	11	3	3	-	2	-	-	-	4	16.87	.08–65
NEF	1	5	2	2	-	-	2	-	-	-	-	28.70	6–70
SCH	-	1	-	-	-	-	-	-	-	-	1	*	*
MSA	-	1	-	-	-	-	-	-	-	-	-	*	*
CEM	-	-	2	-	-	-	-	-	-	-	-	*	*
CHSY	-	1	-	-	-	-	-	-	-	-	-	*	*
REBA	1	-	-	-	-	-	-	-	-	-	-	*	*

TOTAL	448	232	372	251	176	155	95	32	7	2	93		

The site distribution of some selected neoplasms is as summarized in table 4. The neoplasms had different site predilections. For instance 55.72% of neoplasms whose site was recorded as the mandible were ameloblastoma while 56.4% of the palatal cases were Kaposi's sarcoma. Unfortunately, in over 50% of all the slips, the site of biopsy wasn't recorded.

**Table 4:** Site distribution of some selected oral facial neoplasms in Uganda and Tanzania

	AME	MYX	FIB	BUR	SCCa	PDCa	PLA	ADN	ACCa	KS	PAP
Maxilla	1	6	26	50	-				1	1	
Mandible	74	1	22	20	4				1		
Jaws	6	-	2	7	1						
Tongue	-	-	7	-	39	1			1	54	13
Palate	-	-	3	-	6	5	26	2	3	97	3
Buccal	-	-	5	-	15			2		13	1
Gingiva	-	-	-	-	3					21	3
Labial	-	-	2	-	7	2	3	2	2	13	4
Commisure	-	-	3	-	-						2
Retro molar	-	-	1	-	3	1				5	
Tuberosity	-	-	-	-	1					3	
Parotid	-	-	-	-	-	1	12	1	1	1	
Submandibular	-	-	-	-	-	1	6	2	1		
Sublingual	-	-	-	-	-		1				
Oral cavity	-	-	-	-	3						
Mouth floor	-	-	-	-	21					2	1
Face	-	-	1	-	4					1	
Neck	-	-	-	-	1						
Maxillary antrum	-	-	-	-	4						
Nassal area	-	-	-	-	-						
Unspecified	91	14	65	305	171	8	44	9	9	199	30

Total	172	21	138	382	283	19	92	19	19	409	57

## Discussion

In this study, malignant maxillofacial neoplasms accounted for up to 67.28% a figure that is very high compared to other reports. [1,3,5] However, the other reports included cysts, granulomas and inflammatory conditions and thus malignancies expressed as a percentage of all the biopsies were bound to be lower. A high frequency of malignancies has been reported by some African studies such as Chidzonga et al and Aregbesola et al compared to Asian, American or European reports. [2-5,13] This could be due to differences in clinical practice whereby in some centers all surgical specimens are sent out for histological investigations, while in others radiographic and clinical features are used as criteria for what needs histopathological investigation. In our study, the exclusion of non neoplastic cysts, hyperplastic and inflammatory lesions combined with the clinical practice of selective histological investigations and high likelihood of referral in case of malignancy, may explain the high frequency of malignancies reported. We decided to leave out the inflammatory and hyperplastic lesions since in many instances they are recorded as no cancer seen which made it difficult to include them in the study. Final diagnosis such as no cancer seen may be due to reliance on already over-stretched general pathologists to diagnose oral pathological slides. Tay et al encountered a similar problem when it came to oral histology reports from general pathologists. [3] The commonest malignancy in these series was Kaposi's sarcoma (21.98%) a neoplasm not reported by Tay et al, Krutchkoff et al, Ogunbodede et al. [3,13,14] Jones et al reported KS lesion (0.3%) at a UK center but the percentage was low compared to our study. [5] This may be due to the fact that KS biopsies are obtained from the skin in other centers. On the other hand Mbulaiteye SM et al reported an increased standardized incidence risk for KS among HIV infected persons of 6.4 (95% CI 4.8–8.4) and therefore many of these biopsies are likely to have been from HIV infected individuals. Additionally Goedert et al earlier on reported a 310 fold increase in risk of acquiring KS following HIV infection while Ziegler et al reported a 40 fold increment with the oro-facial dominant pattern accounting for up to 79% of the cases in Uganda. [15-17] In this study Uganda contributed the bulk of Kaposi's sarcoma cases as shown in table 1, hence Ziegler et al's report may be a good explanation for this unusually high frequency of KS in our series. However, it is worth noting that this is highly speculative since we don't have the HIV status of the cases diagnosed with Kaposi's sarcoma. Presently the picture may be different given the increased availability of highly active antiretroviral therapy (HART), which have been reported to decrease the prevalence of some AIDS-associated oral facial conditions. [18,19]A recent study in Tanzania noted a very low prevalence of Kaposi's sarcoma among patients on HART. [20] It would be interesting to do research on the trend of Kaposi's sarcoma with the advent of HART versus the period prior. There was equal sex distribution of KS (Table 2), suggesting that the exposure to risk factors is equal. The majority of the KS cases (69.68%) were in the 3^rd ^and 4^th ^decade (Table 3). This tallies with the HIV AIDS prevalence that is highest in the 3^rd ^and 4^th ^decades. [21,22] The palate and tongue were the commonest sites for KS lesions (47.55%, 26.47% respectively) however, more than half of the biopsy reports, the site was not specified. The gender distribution of 1:1 was similar to that reported by earlier studies. [15,16]

The second commonest malignancy overall was Burkitts lymphoma (20.54%). However, in Tanzania, Burkitt's lymphoma came in third after squamous cell carcinoma (Table 1). Burkitt's lymphoma has been consistently reported at high frequencies in African series but not Asian and American series. [3,4,12,23,24] This has lead to speculations as to why such a high frequency occurs in Africa. The various reasons advanced include geographical, HIV/AIDS, socio economic factors, nutrition, viral infections and genetic. [14,16,25,26] In our study it's hard to explain the high frequency hence more studies are required to investigate whether the improving situation in African countries is showing reduction in this tumor to levels close to those seen in other parts of the world. A recent study from Kenya reported geographical differences in clinical features of Burkitt's lymphoma. The coastal, western and central regions had facial swelling as the presenting complaint in 81%, 64% and 31% respectively. [27] Such a phenomenon could lead to a high number of Burkitts lymphomas in oral facial biopsies in some centers compared to others and may as well explain the huge numbers in Uganda compared to Tanzania and absence of Burkitt's lymphoma in some studies from Africa. The gender distribution was similar to other reports from Nigeria and Kenya with boys being more affected than girls (table 2). [4,12,27] The mean age at diagnosis was 7.21 ± 0.513 years with a range of 2–45 (table 3). It is worth noting that in this study 89.3% were under the age of 10 and 9.2% between 10 and 20 years. The frequency distribution of those below 10 is as shown in figure 1. These findings agree with other reports from other parts of Africa.[4,12,24] The site of Burkitt's lymphoma was recorded in only77 cases (mandible 65%, the jaws i.e. both maxilla and mandible accounted 9.1% and maxilla 14.9%). Although very few cases had the site recorded, the results are in agreement with already published reports. [4,12] There were 17 cases reported as non Hodgkin's lymphoma of which only two were below 10 years and 8 over 40 years. These could have been any other types of non hodgkins lymphoma such as large cell lymphoma. Jones et al [5] had non Hodgkin's lymphoma ranked as second commonest malignancy in their study.

The third commonest malignancy was squamous cell carcinoma accounting for 15.22% overall. This was different when compared to reports from both other African studies and the rest of the world that report squamous cell carcinoma as the most frequent malignancy. [1-3,14] However, it should be noted that the differences in inclusion criteria, clinical practices make it impossible to objectively compare these findings with others. For instance neither the Asian and American studies report of any case of Burkitt's lymphoma nor does the Jordanian study despite the fact that it was based on children and adolescents. [1,3,23]. The difference between our study and those from Zimbabwe [2] and Nigeria [14] could be due to the high number of KS cases that were not included in those two studies. When we considered only the tumors in the Nigerian study, squamous cell carcinoma prevalence in Tanzania increased to nearly equal levels as those reported in Nigeria but in Uganda the high number of Burkkit's lymphoma cases still pulled down the prevalence of squamous cell carcinoma. This has been reported in a different Nigerian study hence giving some credence to our argument. [28] The gender and age distribution are shown in tables 2 and 3. The Mean age didn't differ from that of the individual countries. The modal age group in both hospitals was 51–60 (p ≥ 0.01). This is in line with what was expected since squamous cell carcinoma is known to mainly affect older individuals. However, interestingly in Tanzania males were more affected which is in line with most parts of the world but in Uganda there was equal sex distribution. Some studies from other parts of the world have already noted an increase in oral cancer incidence among females but stable rates among men. [28-31]This is attributed to equal exposure to cancer risk factors between women and men and probably over time Tanzania will register a similar trend as Uganda in terms of equal prevalence among women and men. Unfortunately the Kenyan study didn't report on squamous cell carcinoma to allow comparison. [12]The tongue, floor of the mouth and buccal surface were the commonest sites among those specified. The tongue as a site of head and neck cancer ranked high in Nigeria and the incidence of lingual squamous cell carcinoma has been reported to be on the increase in America. [28,32] It's worth noting that unlike the Nigerian study from Ile Ife where no cancer of the buccal mucosa was reported, it was the third commonest site in our study. [14] On the other hand a study from Zimbabwe had similar trends in anatomical sites with ours. [33] Therefore more research is needed to see if there are differences in anatomical sites affected by squamous cell carcinoma across Africa compared to the rest of the world. Anaplastic carcinoma and undifferentiated/poorly differentiated carcinoma accounted for 1.88% of all malignant neoplasms seen. This is similar to what has been reported by Jones et al. [5]

There was only one case of malignant odontogenic neoplasm in these series, which showed the rarity of these entities.

Salivary gland neoplasia is one of the challenging tumors when it comes to histological typing. The WHO classification is aimed at making it easier for researchers to compare prevalence, however, not all centers have re-diagnosed the entity in accordance with it. In this study salivary gland neoplasia accounted for about 9% of both malignant and benign neoplasms, a figure close to that reported from Europe. [5] Pleomorphic adenoma dominated this group. The peak frequency was the 3^rd ^decade with equal gender distribution, however, reports from Brazil and Italy showed a female preponderance.[5,34-36] Multi center studies are needed to see if there is real sex predilection or not. Adenoma was the second commonest benign salivary gland neoplasm. This entity is not included in the WHO classification [37] nor is it reported by Jones et al and Lima et al.[5,35] These could be one of the other adenomas such as canalicular and monomorphic adenoma that were not sub-typed. There is need for an experienced oral pathologist to review these slides so as to bring them inline with the WHO classification. Interestingly there was no report of Warthins tumor in our findings an entity reported by others. [5,35] Since this entity has been reported else where in Africa [38], we cannot explain its complete absence in our series and may be a review of slides could yield a few. Among the malignant salivary gland neoplasms, adenocarcinoma was the commonest which is in line with Jones et al but different from Lima et al. However it should be noted that Jones et al reported adenocarcinoma and salivary gland adenocarcinoma as independent entities yet in our study and that of Lima SS et al there was no distinction between the two. This could point to the need for a multi-center study to reclassify these neoplasms as per WHO standing nomenclenture. Of the benign neoplasms recorded in our study, 33% were odontogenic with ameloblatoma accounting for 84% of this group. This was in agreement with African and Chinese studies but differed from European and American studies in which odontomas dominated the odontogenic neoplasms.[1,2,5-7,11,12,39] In fact only one case of odontoma was found in this series. The high frequency of ameloblastoma in our study compared to Europe and America may as well be due to different clinical practices such as not sending all specimens for hitopathological evaluation, the inclusion criteria of different studies and the harvest period. Therefore it could be that odontomas due to their appearance radiologically, surgically and clinical course are not routinely treated and if treated are rarely sent for histology. All the demographics in our study agreed with previous reports.

Among the benign non odontogenic neoplasms including salivary gland neoplasms, fibromas, pleomorphic adenoma and papillomas were the commonest in descending order. This differs from the report by Jones etal [5] in which papillomas then pleomorphic adenomas were ranked as first and second. This is hard to draw conclusions. Both fibromas and papillomas had a slight predilection for females which is in contrast with Jones etal. It should be noted that the numbers in our study are small compared to Jones et al hence this could be a chance finding. The site distribution of these neoplasms was varied as can be seen in table 4. Unfortunately, in the majority of the lesions, the sites were not specified.

Lipomas were also relatively frequent with the demographics all being in line with published reports. In our study hamartomas were not classified as odontogenic or non odontogenic yet they are known to occur under both categories. (40)We included them in the non odontogeinc neoplasms.

## Conclusion

The range of diagnosed neoplasms from our study was diverse and our results should be of interest to pathologists, oral and maxillofacial surgeons, specialist practitioners and general dental practitioners. The results do not represent the actual prevalence of oral and maxillofacial disease within the general population, but simply reflect the frequency of histologically diagnosed neoplasms at Mulago and Muhimbili national referral hospitals of Uganda and Tanzania respectively. This survey has shown that most diagnoses are malignant in nature and often require surgical management. However, a large proportion of cases such as odontomas, e.t.c., which are not as aggressive, could have been under represented because of their clinical course and the clinical practices at these centers. The disparities noted between the WHO classification and our report should hopefully generate interest to reclassify oro-facial neoplasms across the world to enable better comparison.

## Competing interests

The authors declare that they have no competing interests.

## Authors' contributions

KA Conceived, collected the data and co-wrote the article

KB Co-wrote the article

## Pre-publication history

The pre-publication history for this paper can be accessed here:


